# Cytoreductive prostatectomy improves survival outcomes in patients with oligometastases: a systematic meta-analysis

**DOI:** 10.1186/s12957-022-02715-x

**Published:** 2022-08-09

**Authors:** Yifeng Mao, Mingqiu Hu, Gaowei Yang, Erke Gao, Wangwang Xu

**Affiliations:** 1 Department of Urology, The Second Affiliated Hospital of Bengbu Medical College, Bengbu, Anhui China; 2 Anhui Province Key Laboratory of Translational Cancer Research, Bengbu Medical University, Bengbu, 233030 Anhui China; 3 Department of Urology, Maoming People’s Hospital, Maoming, 525000 Guangdong China

**Keywords:** Cytoreductive prostatectomy, Oligometastases, Overall survival, Cancer-specific survival, Progression-free survival

## Abstract

**Background:**

Whether cytoreductive prostatectomy (CRP) should be performed in patients with oligometastatic prostate cancer (OPC) remains controversial. The goal of this systematic meta-analysis was to assess the efficacy of CRP as a treatment for OPC.

**Methods:**

This systematic review and meta-analysis was conducted in accordance with the Preferred Reporting Items for Systematic Reviews and Meta-Analysis statement. Data sources included publications in the PubMed, Embase, the Cochrane Library, EBSCO, and Web of Science (SCI) databases as of May 2022. Eligible articles included prospective studies comparing the efficacy of CRP to a lack of CRP in patients with OPC.

**Results:**

In total, 10 publications incorporating 888 patients were analyzed. Tumor-reducing prostatectomy was found to have no significant effect on long-term or short-term OS [*OR* = 2.26, 95% *CI* (0.97, 5.28), *P* = 0.06] and [*OR* = 1.73, 95% *CI* (0.83, 3.58), *P* = 0.14], but it significantly improved patient long-term or short-term CSS [*OR* = 1.77, 95% *CI* (1.01, 310), *P* = 0.04] and [*OR* = 2.71, 95% *CI* (1.72, 4.29), *P* < 0.0001] and PFS [*OR* = 1.93, 95% *CI* (1.25, 2.97), *P* = 0.003].

**Conclusion:**

These results suggest that cytoreductive prostatectomy can confer survival benefits to OPC patients.

**Trial registration:**

INPLASY protocol 202260017 10.37766/inplasy2022.6.0017.

**Supplementary Information:**

The online version contains supplementary material available at 10.1186/s12957-022-02715-x.

## Background

Prostate cancer is the second most common malignancy in men worldwide, accounting for 3.8% of male cancer deaths in 2018 and making up 191,930 out of the 893,660 new cancer cases diagnosed among US men in 2020 [1, 2]. Roughly, 40–50% of prostate cancer cases are associated with genetic mutations, including newly discovered BRCA1 and BRCA2 mutations and the loss of ATM, which is considered to be a risk factor for the development of this cancer type [3, 4]. While surgery and radiotherapy can be used to treat early-stage prostate cancer, after initial good responses that are maintained for a median of 18 to 24 months, patient prostate-specific antigen (PSA) levels continue to rise, culminating in the development of metastatic castration-resistant prostate cancer (mCRPC) [5, 6]. Androgen deprivation therapy (ADT) remains a therapeutic mainstay for advanced metastatic prostate cancer patients, with systemic therapy being critical. For example, the CHAARTED trial found that relative to patients treated via ADT alone, the combined administration of docetaxel can improve overall survival (OS) to 10.4 months [7]. However, there is also a growing evidence that radical prostatectomy and stereotactic radiotherapy can afford therapeutic benefits to metastatic prostate cancer patients [8].

Hellman and Weichselbaum were the first to propose oligometastatic disease as an intermediate state between localized primary disease and widespread disseminated metastasis during early-stage tumor radiotherapy treatment [9, 10]. However, international definitions of oligometastases remain inconsistent and controversial, with some studies defining this status based on imaging findings of ≤ 5 metastases including those of the lymph nodes, bones, or vertebrae in the absence of visceral organ metastases [11–13]. The value of local treatment in individuals with metastatic disease has historically been limited by difficulties in locating these metastases, with systemic treatment offering an opportunity to slow the progression of disease and thereby prolong the OS of treated patients. However, subsequent research has shown that cytoreductive surgery offers some therapeutic benefits in certain cancer types including ovarian cancer, metastatic renal cell carcinoma, and pancreatic neuroendocrine tumors [14, 15]. The median OS of patients with metastatic renal cell carcinoma (RCC) is reportedly higher than that of non-cytoreductive nephrectomy (CN) patients (17.1 vs 7.7 months) [14]. A mouse model of prostate cancer has also been found to exhibit reduced metastatic disease progression and prolonged survival following cytoreductive surgery [16]. Recent evidence has further supported the benefit of primary tumor resection in mice with metastatic prostate cancer, with treated animals surviving for longer, exhibiting slower rises in PSA levels, and presenting with fewer pulmonary metastases [17]. Despite such evidence, the value of tumor-reducing surgery in prostate cancer patients remains the subject of controversy.

While there is growing consistency among many studies, full consensus regarding the definition of oligometastatic prostate cancer is still lacking. At the 2019 APCCC meeting [18], there was considerable disagreement regarding the location of the metastases in such cases, with ~46% of panelists voting for a definition entailing a limited number of synchronous or metachronous metastases in the bone or lymph nodes, but not for metastases affecting the internal organs, while 33% supported a definition including a limited number of synchronous or metachronous metastases including visceral organ metastases, 8% supported a definition including a limited number of bone or lymph node metachronous metastases in the absence of visceral organ metastases, 4% supported a definition including a limited number of metachronous metastases and metastatic disease including visceral organ metastasis, and 9% believed that oligometastatic prostate cancer was not clinically significant. Despite such controversy, clinical consensus is relatively unified with respect to the number of transfers patients should undergo, with 48% of the members having been in favor of three or fewer transfers, while 41% were in favor of five or fewer transfers. Based on analyses of the results of the STAMPEDE trial [19], the HORRAD trial [20], and the STOPCAP meta-analysis [21], 98% of the panel members recommended local treatment of the primary tumor, whereas cytoreductive surgery was not regarded as being effective in patients with oligometastatic prostate cancer. As such, the overall benefit of such treatment remains highly debated.

The present study was therefore developed to explore the value of cytoreductive surgery in oligometastatic prostate cancer patients by pooling data from published prospective studies and conducting a comprehensive systematic review and meta-analysis in which patient OS, cancer-specific survival (CSS), and progression-free survival (PFS) were systematically analyzed to gauge the benefits of this therapeutic approach.

## Materials and methods

The protocol for this systematic review was registered on INPLASY (Unique ID number: INPLASY202260017) and is available in full on inplasy.com (https://inplasy.com/inplasy-2022-6-0017/). This analysis was performed in accordance with the Preferred Reporting Items for Systematic Reviews and Meta-Analyses (PRISMA) statement.

### Patient and public involvement

Patients and the public had no role in the design or execution of this study.

### Study selection

The PubMed, Embase, Cochrane Library, EBSCO, and Web of Science (SCI) databases were searched for all relevant studies published as of May 2021 using MeSH terms and free-text terms including the following: prostate cancer, oligometastatic OR oligometastasis OR oligometastases, and prostatectomy OR cytoreduction surgical procedures. The references of relevant studies were also manually reviewed to identify other studies of interest. Only studies published in English were included in this meta-analysis, which was conducted in accordance with PRISMA guidelines [22]. Studies eligible for inclusion met the following criteria: (1) studies of patients with oligometastatic prostate cancer, as defined by the presence of ≤ 5 metastases, (2) studies examing the clinical outcomes associated with cytoreductive surgery in oligometastatic prostate cancer patients, and (3) studies reporting relevant outcomes following surgery including OS, CSS, and/or PFS.

**Fig. 1:**
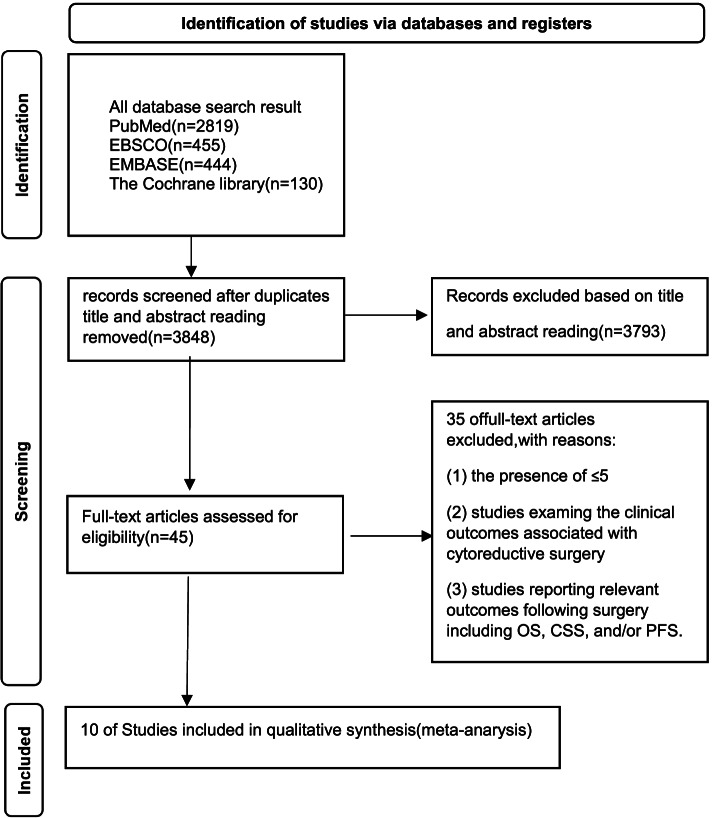
Search strategy flow diagram

Two researchers (YM and GW) separately identified relevant studies and extracted data therefrom, with disagreements being resolved through discussion and consensus with a third researcher (MH). The study selection process is outlined in Fig. 1.

### Study quality assessment

The Newcastle-Ottawa scale (NOS) was utilized to examine prospective cohort study quality [23, 24]. The NOS is considered the most extensive approach to evaluating randomized controlled trials. All included studies scored 6 points or higher on this scale and were thus considered to be of high quality (Table 1).

**Table 1:** The characteristics of included studies for meta-analysis

Authors and year	Patients	Group	No of patients	Ag(range)(years)	Follow-up(range)(mo)	CPFS	CSS	OS
Grimm MO al. 2002 [25]	82	RP	50			53%(5-y) 36%(10-y)	90%(5-y) 47%(10-y)	86%(5-y) 34%(10-y)
		ADP	32			31%(5-y)10 15%(10-y)	53%(5-y) 32%(10-y)	39%(5-y) 17%10-y)
Thomas Steuber al. 2011 [26]	158	RP-	50	62(49-70)	98(88-113)	61%(5-y) 31%(10-y)	81%(5-y) 46%(10-y)	80%(5-y) 42%(10-y)
		RP+	108	64(46-76)	98(88-113)	77%(5-y) 61%(10-y)	84%(5-y) 76%(10-y)	79%(5-y) 69%(10-y)
Axel Heidenreich al. 2015 [27]	61	ADT	38	63.9(47-83)	44.0 (24-96)		84.2%(3-y)	78.9%(3-y)
		CRP	23	61(42-69)	40.6 (3-71)		95.6%(3-y)	91.3%(3-y)
Bimal Bhindi al. 2017 [28]		RRP	34				79%(5-y)	77%
		-RRP	34				55%(5-y)	55%
M Moschini al. 2016 [29]	61	Surgery	31	62 (56–66)	38.8		100%(1-y) 91.3%(3-y) 61.9%(5-y)	
		ADT	16	59 (54–59)			93.8%(1-y) 76.9%(3-y) 46.2%(5-y)	
Ming-Xiong Sheng al 2017 [30]	49	Surgery	23	68.1 ± 9.9 (57–83)	37(19-53)	86.96(5-y)		86.96%(5-y)
		ADT	26	72.0 ± 4.7 (63–84)	41(24-56)	92.31%(5-y)		73.08%(5-y)
Won Sik Jang al. 2018 [31]	79	ADT	41	71 (67−76)	40(25-48)	41/41(1-y) 17/41(3-y) 4/41(5-y)	40/41(1-y) 16/41(3-y) 6/41(5-y)	
		RARP	38	65 (62−69)	40(25-48)	37/38(1-y) 24/38(3-y) 14/38(5-y)	38/38(1-y) 31/38(3-y) 14/38(5-y)	
Tian Lan al. 2019 [32]	111	ADT	76	71.17 ±7.73	35(22-41)	27.0%(3years) 21.0%(5years)	87.9%(3years) 74.9%(5years)	85.5%(3-y)
		CRP+ADT	35	67.83± 7.19	35(25-45)	42.7%(3years) 19.0%(5years)	90.8%(3years) 63.6%(5years)	88.6%(3-y)
Nasser Simforoosh al. 2019 [33]	49	ST	23		22.8(14-43)		65.2%(3-y)	65.2%(3-y)
		CRP	26		19.2(9-42)		76.9%(3-y)	76.9%(3-y)
Shubin Si al. 2021 [34]	84	RP	27	76.67 ± 9.66				96.2%(3-y) 76.0%(5-y)
		-RP	57	76.42 ± 9.69				94.7%(3-y) 74.9%(5-y)

Continuous variables were expressed as (mean±SD), mean (range) or median (IQR)

*PSA* The nadir of prostate-specific antigen, *CSS* Cancer-specific survival, *OS* overall surviva, *RP* radical retropubic prostatectomy, *CRP* cytoreductive prostatectomy, *RARP* robot-assisted radical prostatectomy, *ADT* androgen deprivation therapy

### Data extraction and statistical analysis

Posttreatment outcomes of interest including OS, CSS, and PFS were extracted from included studies. The pooled data were expressed in the form of risk odds ratios (ORs) and 95% confidence intervals (CIs). The *I*^2^ statistic was used to assess heterogeneity among studies, with *I*^2^ < 50% being indicative of acceptable heterogeneity. When heterogeneity was acceptable, results were analyzed with a fixed-effects model, whereas a random-effects model was otherwise used. The *Z*-test was used to analyze pooled effects, with *P* < 0.05 as the significance threshold.

### Sensitivity analysis

The reliability of results was assessed through sensitivity analyses for the OS, CSS, and PFS endpoints at 3- and 5-year time points. The Review Manager v5.4 software was used for all data analyses.

## Results

In total, 10 relevant studies [25–34] incorporating 888 patients were included in the present analysis after having met with study selection criteria (Table 1). All included prospective studies were considered to be of high quality (Table 2). All 10 articles defined metastases in oligometastatic prostate cancer as five or fewer metastases, and all control patients were treated with ADT, with no suspicious visceral involvement being observed upon pretreatment imaging. One study employed robotic surgery approaches, and one study employed cryosurgery approaches for patient treatment.

**Table 2:** Evaluation of study quality

Study	Selection				Comparability	Exposure			Scores
	Adequate definition of cases	Representativeness of the cases	Selection of controls	Definition of controls	Control for important factor	Ascertainment of exposure	Same method of ascertainment for cases andcontrols	Non-response rate	
Grimm MO al. 2002 [25]	☆	☆	☆	☆	/	☆	☆	☆	7
Thomas Steuber al. 2011 [26]	☆	☆	☆	☆	/	☆	☆	☆	7
Axel Heidenreich al. 2015 [27]	/	☆	☆	☆	☆	☆	/	☆	6
Bimal Bhindi al. 2017 [28]	/	☆	☆	☆	☆	☆	☆	☆	7
M Moschini al. 2016 [29]	☆	☆	☆	☆	☆☆	☆	/	☆	8
Ming-Xiong Sheng al 2017 [30]	/	☆	☆	☆	/	☆	☆	☆	6
Won Sik Jang al. 2018 [31]	☆	☆	☆	☆	☆	☆	/	☆	7
Tian Lan al. 2019[32]	☆	/	☆	☆	☆	☆	/	☆	6
Nasser Simforoosh al. 2019[33]	☆	/	☆	☆	/	☆	☆	☆	6
Shubin Si al. 2021[34]	☆	☆	☆	☆	☆☆	☆	/	☆	8

### Overall survival

**Fig. 2:**
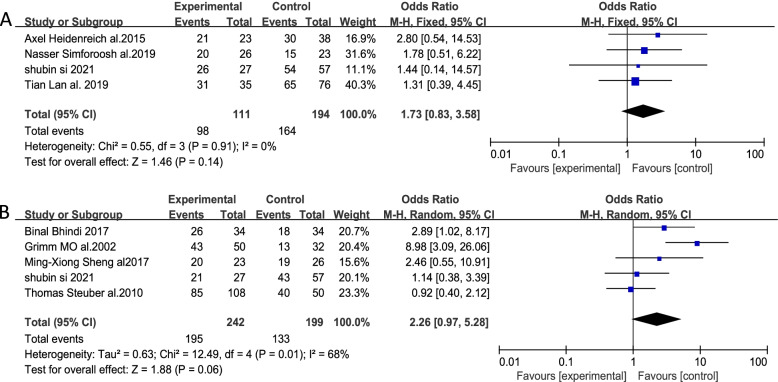
Forest plot corresponding to patient overall survival at 3 years (**A**) and 5 years (**B**) in the cytoreductive prostatectomy group and the androgen deprivation therapy group. No significant difference in OS was observed at 3 years (**A**) [*OR* = 1.73, 95% *CI* (0.83, 3.58), *P* = 0.14 > 0.05] or 5 years (**B**) [*OR* = 2.26, 95% *CI* (0.97, 5.28), *P* = 0.06 > 0.05]

Of the included studies, 8 reported patient OS, including 4 that reported 3-year OS outcomes. Data were analyzed using a fixed-effects model (*I*^2^ = 0%, *P* = 0.91). There was no significant difference between the experimental and control groups [*OR* = 1.73, 95% *CI* (0.83, 3.58), *P* = 0.14] (Fig. 2A). Additionally, 5 studies reported patient 5-year OS, and the results were analyzed with a random-effects model owing to the presence of heterogeneity (*I*^2^ = 68%, *P* = 0.01). There were no significant differences between the experimental and control group in this analysis [*OR* = 2.26, 95% *CI* (0.97, 5.28), *P* = 0.06] (Fig. 2B). These data indicated that tumor reduction surgery failed to improve patient OS.

### Cancer-specific survival

**Fig. 3:**
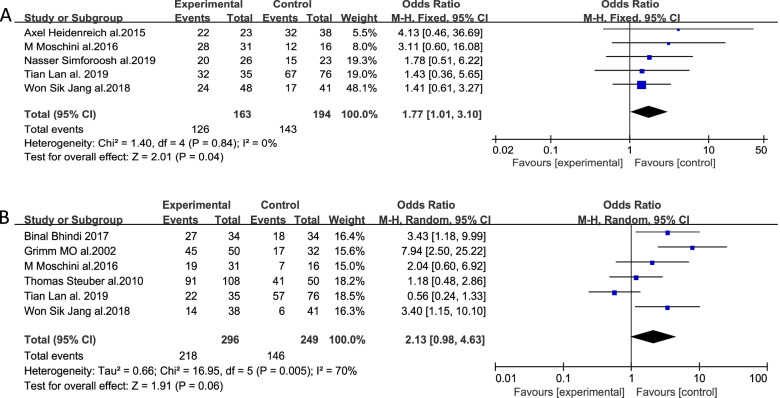
Forest plot corresponding to patient CSS at 3 years (**A**) and 5 years (**B**) in the cytoreductive prostatectomy group and the androgen deprivation therapy group. Patients in the group that underwent surgery exhibited significantly higher CSS at 3 years (**A**) [*OR* = 1.77, 95% *CI* (1.01, 3.10), *P* = 0.04 < 0.05] but not at 5 years (**B**) [*OR* = 2.71, 95% *CI* (0.98, 4.63), *P* = 0.06 > 0.05]

In total, 8 of the included studies reported on patient CSS, of which 5 reported patient 3-year CSS outcome data. Results were analyzed with a fixed-effects model (*I*^2^ = 0%, *P* = 0.84), revealing a significant difference between the surgery and non-surgery groups [*OR* = 1.77, 95% CI (1.01, .10), *P* = 0.04] (Fig. 3A). Moreover, 6 studies reported patient 5-year CSS. There was significant heterogeneity associated with this endpoint (*I*^2^ = 70%, *P* = 0.005), with results thus being analyzed using a random-effects model. There were no significant differences between the experimental and control group in this analysis [*OR* = 2.71, 95% *CI* (0.98, 4.63), *P* = 0.06] (Fig. 3B). As such, tumor reduction surgery is associated with significant improvements in 3-year but not 5-year CSS.

### Progression-free survival

**Fig. 4:**
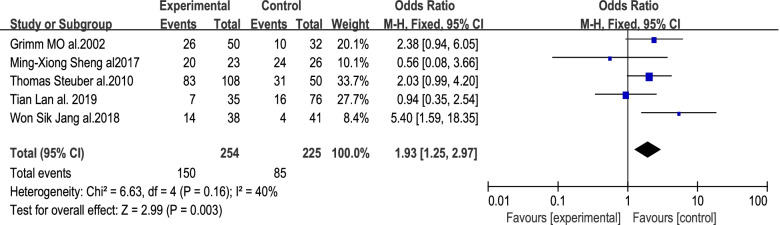
Forest plot corresponding to patient PFS at 5 years in the cytoreductive prostatectomy group and the androgen deprivation therapy group. A significant difference in 5-year PFS was observed between the surgery and non-surgery groups, with survival being significantly longer for patients that underwent surgery [*OR* = 1.93, 95% *CI* (1.25, 2.97), *P* = 0.003 < 0.05]

In total, 5 studies reported on 5-year PFS outcomes for included patients, with data being analyzed using a fixed-effects model (*I*^2^ = 40%, *P* = 0.16). A significant difference in 5-year PFS was observed between the surgery and non-surgery groups [*OR* = 1.93, 95% *CI* (1.25, 2.97), *P* = 0.003] (Fig. 4), thus indicating that tumor reduction surgery can significantly improve patient 5-year PFS.

### Sensitivity analyses

Sensitivity analyses were performed for each of the five analyzed outcomes. For these analyses, individual studies were iteratively excluded from the corresponding outcome assessments to examine the effect of the absence of a given study on overall result stability. The results pertaining to 3-year OS, 3-year CSS, and 5-year PFS outcomes remained consistent in these sensitivity analyses, confirming the reliability of the results. However, the results for the 5-year CSS and 5-year OS outcomes were altered when the studies conducted by Steuber et al. [26] and Lan et al. [32] were excluded, respectively, consistent with the presence of heterogeneity that affected the above results. However, as no more than 10 studies were included in these analyses, these skewed results may have been inaccurate and represent a potential source of publication bias.

## Discussion

Prior reports have indicated that cytoreductive surgical treatment of primary tumors can afford benefits to the survival and quality of life of patients with certain cancer types. For example, Rapiti et al. demonstrated that tumor reduction surgery improves overall survival in patients with metastatic breast cancer [35–37]. This has led some researchers to propose a “seed and soil” theory in which primary tumor cells can act as circulating tumor cells (CTCs) that seed both local and distal metastatic tumor growth [36, 37]. Sheng et al. have demonstrated that CTCs can be used as a prognostic and therapeutic response marker for prostate cancer [38]. As such, prolonged primary tumor survival may increase the odds of further disease metastasis [35–37, 39, 40].

In certain diseases including ovarian cancer, metastatic renal cell carcinoma, and pancreatic neuroendocrine tumors, the benefits of primary tumor cytoreductive surgery have been confirmed. There is also further evidence that tumor reduction can improve quality of life for oligometastatic prostate cancer patients [41, 42]. Importantly, this surgical intervention is feasible and safe in individuals with metastatic prostate cancer. However, as randomized controlled trials focused on this surgical intervention in oligometastatic prostate cancer patients are lacking, its purported survival benefits remain controversial.

The meta-analysis published by Cheng et al. demonstrated that cytoreductive surgery was associated with obvious advantages in terms of overall survival, tumor-specific survival, and progression-free survival [43]. In contrast, our included studies were more recent (published after 2000) and included more comprehensive and up-to-date data. In addition, rather than simply assessing OS, CSS, and PFS, we examined 3-year and 5-year OS, CSS, and PFS in these patients, which may have contributed to these distinct study findings. Our analysis revealed that cytoreductive surgery can effectively improve the 3-year CSS and 5-year PFS of patients, but cannot improve the overall survival rate or 5-year CSS of patients in the short and medium term. Multiple reports have similarly demonstrated the benefits of cytoreductive surgery in metastatic prostate cancer, as in a study performed by Cul et al. assessing 8185 patients with stage 4 (M1a–c) PCa (NSR (*n* = 7811), RP (*n* = 245)), which found debulking surgery to significantly improve both 5-year OS (67.4% vs 22.5%) and 5-year CSS (75.8% vs 48.7%) in these patients (*P* < 0.01 )[44]. Gratzke et al. also recently analyzed the Munich Cancer Registry dataset and found that of the 1538 newly diagnosed prostate cancer patients included therein, 74 who had undergone RP exhibited significantly higher 5-year survival outcomes as compared to patients that did not (55% vs. 21%) (*P* < 0.01) [45]. Heidenreich et al. further analyzed 113 metastatic prostate cancer patients from 4 institutions who had undergone surgical treatment and observed respective 3- and 5-year OS rates of 87.6% and 79.6%, with 3- and 5-year CSS rates of 89.3% and 80.5%, respectively [46]. As such, cytoreductive debulking therapy offers benefits to the CSS and OS of metastatic prostate cancer patients. However, whether cytoreductive surgery also offers any overall benefit in oligometastatic prostate cancer remains to be confirmed. Using prospective institutional data, Steuber et al. compared 43 patients with oligometastatic prostate cancer treated with CRP and 40 patients that underwent optimal systemic therapy and found that at a median follow-up of 82.2 months, there were no significant differences in CSS (*P* = 0.92) or OS (*P* = 0.25) between these groups [47]. The findings of this study are consistent with our results, suggesting that debulking surgery does not improve the overall survival rate of treated patients.

In one single-institution long-term analysis of 11 oligometastatic prostate cancer patients, Gandaglia et al. reported 7-year clinical progression and cancer-specific mortality (CSM)-free survival rates of 45% (95% *CI*, 30–85%) and 82% (95% *CI*, 62–99%) [48], respectively, with long-term rates of CSM-free survival being higher than those for ADT only (48–55%) [44, 48]. This is inconsistent with the results of our analysis, which may also be due to the short follow-up time in the included studies. However, Battaglia et al. further conducted metastatic surgical treatment in 17 oligometastatic prostate cancer patients and observed a 4-year OS of 66%, with three patients dying of prostate cancer [49]. In addition, Sheng et al. performed statistical analyses of 43 patients and found that cryosurgery prolonged patient PFS by reducing CTC counts [38]. These results and those of our analysis suggest that cytoreductive surgery can significantly improve short-term oligometastatic prostate cancer patient CSS and PFS.

Overall, the results of this meta-analysis suggest that cytoreductive surgery does not improve the OS of prostate cancer patients. This may be attributable to the limited number of included studies and limited overall sample size or may suggest that the side effects associated with cytoreductive surgery may contribute to a lack of overall benefit to patient OS.

There are several limitations to this analysis. For one, as randomized clinical trials exploring this therapeutic approach are lacking, the majority of included studies were retrospective in nature and of varying levels of quality. There were also inconsistencies among studies with respect to the standards used for patient inclusion, and parameters such as PSA levels or age cannot be controlled for in our pooled analyses. Moreover, one of the included literature focused on patients that had undergone cryosurgery, in contrast to open or robotic surgical approaches [30]. Cryosurgery is well tolerated as it is associated with reduced intraoperative blood loss and decreased trauma and has an impact on the overall survival benefits of patients. Our results are inevitably impacted by the short follow-up duration and the limited numbers of patients in the included studies. There was also substantial heterogeneity among these studies with respect to the stage of metastatic prostate cancer patients included in the corresponding analyses, further complicating the interpretation of these results and underscoring directions for further research.

## Conclusion

The results of this meta-analysis suggest that cytoreductive surgery may confer certain survival benefits to prostate cancer patients with oligometastatic disease. However, additional large-scale prospective randomized controlled trials will be essential to validate these results and to establish the overall benefit of such treatment to the quality of life of patients suffering from this form of cancer.

## Supplementary Information

**Additional file 1.** PRISMA_2020_checklist.

**Additional file 2. **Search strategy.

## Data Availability

Not applicable

## Abbreviations

mCRPC: Metastatic castration-resistant prostate cancer
CRP: Cytoreductive prostatectomy
OPC: Oligometastatic prostate cancer
ADT: Androgen deprivation therapy
OS: Overall survival
CSS: Cancer-specific survival
PFS: Progression-free survival
SCI: Web of Science
CIs: Confidence intervals
CSM: Cancer-specific mortality
CTCs: Circulating tumor cells
