# Supplementary material for: Cytoreductive prostatectomy improves survival outcomes in patients with oligometastases: a systematic meta-analysis
Source: World J Surg Oncol. 2022 Aug 9;20:255. doi: 10.1186/s12957-022-02715-x (PMC9361652; doi:10.1186/s12957-022-02715-x)
Supplement: Supplementary file 2 — Additional file 2. Search strategy. [file 12957_2022_2715_MOESM2_ESM.docx]

**Table S1 Literature search criteria for PubMed and Medline**

| (("Neoplasm Metastasis"[MeSH Terms] OR (("metastasation"[All Fields] OR "metastasic"[All Fields] OR "metastasing"[All Fields] OR "metastasise"[All Fields] OR "metastasised"[All Fields] OR "metastasises"[All Fields] OR "metastasising"[All Fields] OR "metastasization"[All Fields] OR "metastasizes"[All Fields] OR "metastasizing"[All Fields] OR "Neoplasm Metastasis"[MeSH Terms] OR ("Neoplasm"[All Fields] AND "Metastasis"[All Fields]) OR "Neoplasm Metastasis"[All Fields] OR "metastase"[All Fields] OR "Metastases"[All Fields] OR "metastasize"[All Fields] OR "metastasized"[All Fields]) AND "Neoplasm"[Title/Abstract]) OR "neoplasm metastases"[Title/Abstract] OR "Metastasis"[Title/Abstract] OR "Metastases"[Title/Abstract] OR "metastasis neoplasm"[Title/Abstract] OR "Lymphatic Metastasis"[MeSH Terms] OR "lymphatic metastases"[Title/Abstract] OR "metastases lymphatic"[Title/Abstract] OR "metastasis lymphatic"[Title/Abstract] OR "lymph node positive"[Title/Abstract] OR "lymph node metastasis"[Title/Abstract] OR "bone metastasis"[Title/Abstract] OR (("skeletal"[All Fields] OR "skeletals"[All Fields]) AND ("metastasation"[All Fields] OR "metastasic"[All Fields] OR "metastasing"[All Fields] OR "metastasise"[All Fields] OR "metastasised"[All Fields] OR "metastasises"[All Fields] OR "metastasising"[All Fields] OR "metastasization"[All Fields] OR "metastasizes"[All Fields] OR "metastasizing"[All Fields] OR "Neoplasm Metastasis"[MeSH Terms] OR ("Neoplasm"[All Fields] AND "Metastasis"[All Fields]) OR "Neoplasm Metastasis"[All Fields] OR "metastase"[All Fields] OR "Metastases"[All Fields] OR "metastasize"[All Fields] OR "metastasized"[All Fields])) OR "osseous metastasis"[Title/Abstract] OR "bone metastases"[Title/Abstract] OR "Oligometastasis"[Title/Abstract] OR "oligometastases"[Title/Abstract]) AND ("Prostatectomy"[MeSH Terms] OR "Cytoreduction Surgical Procedures"[MeSH Terms] OR "Transurethral Resection of Prostate"[MeSH Terms] OR "local treatment"[Title/Abstract]) AND ("Prostatic Neoplasms"[MeSH Terms] OR "prostate neoplasms"[Title/Abstract] OR "neoplasms prostate"[Title/Abstract] OR "neoplasm prostate"[Title/Abstract] OR "prostate neoplasm"[Title/Abstract] OR "neoplasms prostatic"[Title/Abstract] OR ("Prostatic Neoplasms"[MeSH Terms] OR ("Prostatic"[All Fields] AND "Neoplasms"[All Fields]) OR "Prostatic Neoplasms"[All Fields] OR ("Neoplasm"[All Fields] AND "Prostatic"[All Fields]) OR "neoplasm prostatic"[All Fields]) OR "prostatic neoplasm"[Title/Abstract] OR "prostate cancer"[Title/Abstract] OR "cancer prostate"[Title/Abstract] OR "cancers prostate"[Title/Abstract] OR "prostate cancers"[Title/Abstract] OR "cancer of the prostate"[Title/Abstract] OR "prostatic cancer"[Title/Abstract] OR "cancer prostatic"[Title/Abstract] OR "cancers prostatic"[Title/Abstract] OR "prostatic cancers"[Title/Abstract] OR "cancer of prostate"[Title/Abstract])) AND (2000:2022[pdat]) |
| --- |

**Table S2 Literature search criteria for Embase**

| 'prostate cancer'/exp OR 'prostate cancer' OR (('prostate'/exp OR prostate) AND ('cancer'/exp OR cancer)) OR 'prostatic neoplasms'/exp OR 'prostatic neoplasms' OR (prostatic AND ('neoplasms'/exp OR neoplasms)) AND 'neoplasm metastasis'/exp OR 'neoplasm metastasis' OR (('neoplasm'/exp OR neoplasm) AND ('metastasis'/exp OR metastasis)) And 'oligometastasis' OR 'oligometastases' AND 'prostatic neoplasms'/exp OR 'prostatic neoplasms' OR (prostatic AND ('neoplasms'/exp OR neoplasms)) |
| --- |

**Table S3 Literature search criteria for Cochrane**

| ((prostate cancer):ti OR ('prostate cancer):ti OR (prostatic neoplasms):ti OR (prostatic neoplasms):ti OR (neoplasm prostatic):ti OR (prostatic neoplasm):ti OR (cancer of prostate):ti OR (prostatic cancers):ti OR (dacomitinib):ti) AND (oligometastasis):ti,ab,kw OR (oligometastases):ti,ab,kw) AND ((Prostatectomy):pt OR (Cytoreduction Surgical Procedures):pt OR (Transurethral Resection of Prostate):ti,ab,kw OR (local treatment):ti,ab,kw) |
| --- |

**Table S3 Literature search criteria for EBSCO**

| ((prostate cancer) OR ('prostate cancer) OR (prostatic neoplasms) OR (prostatic neoplasms) OR (neoplasm prostatic) OR (prostatic neoplasm) OR (cancer of prostate) OR (prostatic cancers) OR (dacomitinib)) AND (oligometastasis) OR (oligometastases)) AND ((Prostatectomy) OR (Cytoreduction Surgical Procedures) OR (Transurethral Resection of Prostate) OR (local treatment)) |
| --- |
